# Metabonomic analysis of potential biomarkers and drug targets involved in diabetic nephropathy mice

**DOI:** 10.1038/srep11998

**Published:** 2015-07-07

**Authors:** Tingting Wei, Liangcai Zhao, Jianmin Jia, Huanhuan Xia, Yao Du, Qiuting Lin, Xiaodong Lin, Xinjian Ye, Zhihan Yan, Hongchang Gao

**Affiliations:** 1 School of Pharmaceutical Sciences, Wenzhou Medical University, Wenzhou 325035 China; 2 Radiology Department of the Second Affiliated Hospital, Wenzhou Medical University, Wenzhou 325027, China

## Abstract

Diabetic nephropathy (DN) is one of the lethal manifestations of diabetic systemic microvascular disease. Elucidation of characteristic metabolic alterations during diabetic progression is critical to understand its pathogenesis and identify potential biomarkers and drug targets involved in the disease. In this study, ^1^H nuclear magnetic resonance (^1^H NMR)-based metabonomics with correlative analysis was performed to study the characteristic metabolites, as well as the related pathways in urine and kidney samples of *db/db* diabetic mice, compared with age-matched *wildtype* mice. The time trajectory plot of *db/db* mice revealed alterations, in an age-dependent manner, in urinary metabolic profiles along with progression of renal damage and dysfunction. Age-dependent and correlated metabolite analysis identified that cis-aconitate and allantoin could serve as biomarkers for the diagnosis of DN. Further correlative analysis revealed that the enzymes dimethylarginine dimethylaminohydrolase (DDAH), guanosine triphosphate cyclohydrolase I (GTPCH I), and 3-hydroxy-3-methylglutaryl-CoA lyase (HMG-CoA lyase) were involved in dimethylamine metabolism, ketogenesis and GTP metabolism pathways, respectively, and could be potential therapeutic targets for DN. Our results highlight that metabonomic analysis can be used as a tool to identify potential biomarkers and novel therapeutic targets to gain a better understanding of the mechanisms underlying the initiation and progression of diseases.

Diabetic nephropathy (DN), a leading cause of end stage renal disease, is considered a common microvascular complication of diabetic mellitus, with a high risk of atherosclerotic disease and premature death^1^. Previous studies have shown that the pathogenesis of DN is complex and involves multiple factors, including the activation of the sorbitol aldose reductase, increased production of advanced glycation end products, increased activity of protein kinase C, and altered expression of cyclin-dependent kinases^2^. Although several factors are proposed to be involved in the progression of the disease, the systemic and characteristic metabolic alterations involved in the development of DN remain unclear.

Metabonomic analysis is a systems biology approach that provides global metabolic information in biological samples^3^,^4^. This approach has been widely used in the diagnosis and treatment of diabetes^5^,^6^. Our previous studies using metabonomic approach have identified characteristic differences in the metabolite composition of the urine, kidney, and serum samples of STZ-induced type 1 diabetic rats collected at different time points^7^,^8^. Further, analysis of the temporal evolution of key metabolites revealed changes in energy metabolism and methylamine metabolism during the onset and progression of diabetes^9^. Li *et al.* reported that perturbations in the TCA cycle metabolites play a significant role in the pathogenesis of DN in *db/db* mice^10^. Further, Liu *et al.* observed changes in several metabolites along with defective pathways in the serum and urine samples of a nonhuman primate model of DN^11^. However, the global metabolic changes associated with the development of type 2 diabetic mellitus are not well understood.

To comprehensively profile the changes in metabolite levels associated with the progression of DN, we used the *db/db* mouse, a well-established animal model of type 2 diabetes, which develops features of DN and renal damage with age^12^. With the goals of discovering specific biomarkers of disease progression, identifying perturbed metabolic pathways associated with the pathogenesis of the disease, and discovering potential drug targets, the NMR-based metabonomics with correlative analysis was performed to study the metabolic alterations in the urine and renal tissue samples obtained from the *db/db* mice.

## Results

### Clinical biochemistry and histopathology

Morphometric and body fluid parameters determined by biochemical analysis are shown in Table 1. Compared with the *wildtype* mice, the *db/db* mice exhibited hyperglycemia and increase in body weight throughout the experimental period (from 9 weeks to 17 weeks). Urinalysis revealed significantly elevated urinary albumin to creatinine ratio (UACR) and reduced urine creatinine (CRE) in *db/db* mice, indicating renal dysfunction. As reported previously, the UACR levels increased with age in *db/db* mice, suggesting the progression of the DN^10^.

**Figure 1:**
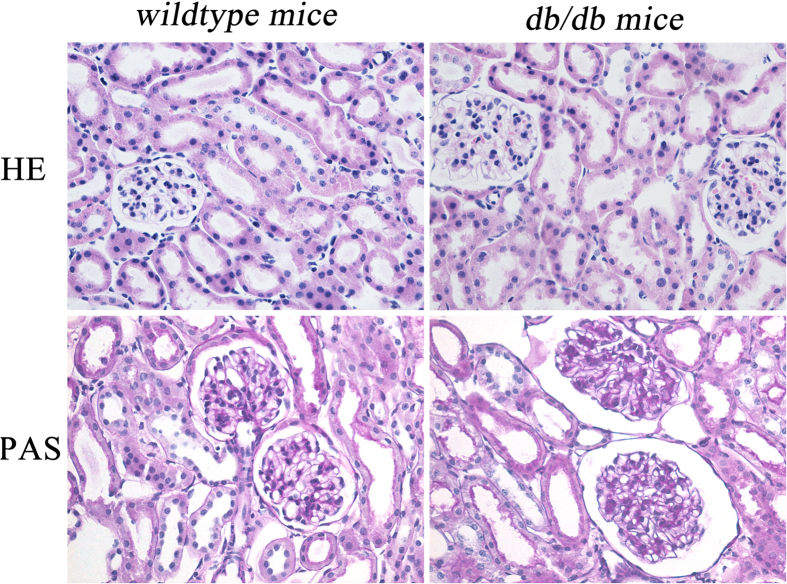
Histological examination of kidney tissues. Representative HE and PAS stains of kidney tissues from 17-week-old *wildtype* mice and *db/db* mice.

Figure 1 shows representative images of the histological examination of hematoxylin-eosin (HE)-stained and periodic acid-schiff (PAS)-stained sections of kidneys from 17-week-old *wildtype* and *db/db* mice. HE staining results revealed prominent glomerular lesions in renal parenchyma at the late stage of DN in *db/db* mice. PAS-stained sections showed clear evidence of glomerulosclerosis. Diffuse mesangial matrix expansion and thickening of the glomerular capillary basement membranes observed in the kidney tissues of 17-week-old *db/db* mice are consistent with previous reports on chronic renal injury in diabetic animals^12^. These results indicated that at the age of 17 weeks the *db/db* mice develop clear manifestations of renal tissue damage and dysfunction.

### NMR spectra and pattern recognition analysis of urine samples

**Figure 2:**
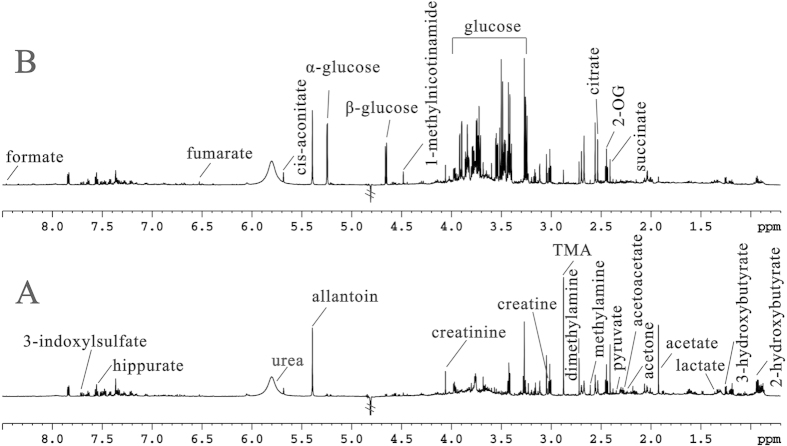
NMR spectra of urine samples. Representative ^1^H NMR spectra of urine samples obtained from the *wildtype* mice (**A**) and *db/db* mice (**B**), respectively.

Figure 2 illustrates typical 600 MHz ^1^H NMR spectra of urine samples obtained from the *wildtype* and the *db/db* mice. The spectral resonances of the metabolites were assigned based on our previous study^9^ and the 600 MHz library of the Chenomx NMR suite 7.0 (Chenomx Inc., Edmonton, Canada). The spectroscopic profiling of the two groups revealed obvious alterations in metabolite concentrations. As expected, compared with *wildtype* mice, the *db/db* mice showed much stronger signal intensity for glucose, indicating glycosuria, a common feature of DN.

**Figure 3:**
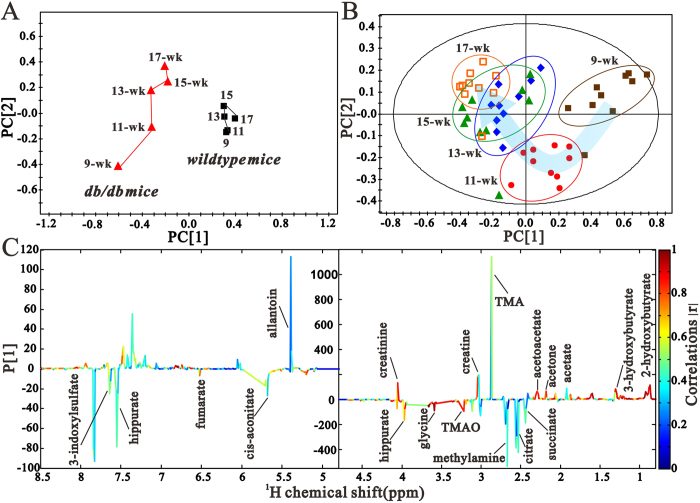
Pattern recognition analysis of urine samples. (**A**) PLS trajectory based on the mean ^1^H NMR spectra of urine samples collected from the *db/db* mice (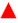) at various time points (9-wk, 11-wk, 13-wk, 15-wk and 17-wk), and the age-matched *wildtype* mice (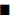). (**B**) The PLS-DA score plot based on ^1^H-NMR spectra of urine samples from *db/db* mice 9-wk (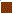), 11-wk (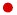), 13-wk (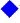), 15-wk (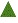) and 17-wk (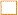). (**C**) is the loading plot revealing the metabolites with large intensities responsible for the discrimination of the corresponding score plot shown (**A**).

To explore the characteristic alterations in urinary metabolites during the progression of DN, urine samples collected at specific intervals from the *db/db* mice and their age-matched *wildtype* controls were analyzed by NMR-based metabonomics. The projection to latent structure discriminant analysis (PLS-DA) mean trajectory showed a characteristic difference in metabolic pattern between the two groups (Fig. 3A). Additionally, results from the *db/db* mice revealed obvious trajectory space from 9 weeks to 17 weeks, while the *wildtype* mice occupied a minor position in the plot, suggesting that the observed changes in metabolic profiles were related to the progression of the disease, but not due to the age of mice. To identify the DN-specific metabolic changes, urinary metabolic trajectory from the *db/db* mice was analyzed (Fig. 3B). As indicated by the arrow, the urine samples of 9-week-old *db/db* mice were distinguishable from that of the 11-week-old mice and the other three samples. The age-dependent metabolic profiles indicated continuous metabolic modifications throughout the experimental period in *db/db* mice, which were manifestations of the pathological insult. Figure 3C illustrates the corresponding loading plot with color-coded correlation coefficients (|r|) of metabolites. The plot showed differences in the levels of metabolites, including 3-HB, acetate, acetone, acetoacetate, succinate, citrate, methylamine, creatine, TMAO, glycine, creatinine, hippurate, allantoin, cis-aconitate, fumarate, and 3-indoxylsulfate.

### Temporal changes in urinary metabolite levels and screening for potential biomarkers

The trajectory plots of urinary metabolic profiles matched with different periods of *db/db* mice, including early stage (9 weeks), middle stage (13 weeks), and late stage (17 weeks) of DN, as defined previously^13^. This difference in metabolite profiles in different age groups of the *db/db* mice can be attributed to the metabolic changes associated with disease progression.

**Figure 4:**
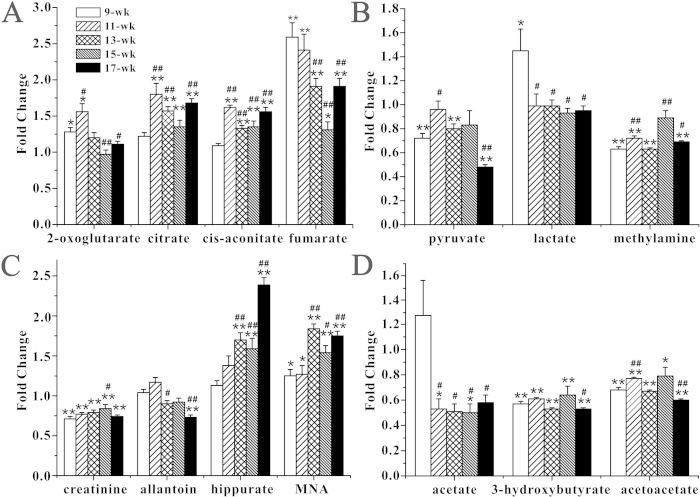
Quantitative analysis of urinary metabolites. Relative abundances of metabolites obtained from ^1^H NMR spectra of urine samples collected from the *db/db* mice and the *wildtype* mice at 9-wk, 11-wk, 13-wk, 15-wk and 17-wk, respectively. Keys: MNA, 1-methylnicotinamide; ^*^P < 0.05 and ^**^P < 0.01 compared with age-matched *wildtype* mice; ^#^*P* < 0.05 and ^##^*P* < 0.01 compared with the *db/db* mice at 9-wk.

The changes in key metabolites from the onset to the progression of the disease are shown in Fig. 4. Compared with the *wildtype* mice, elevated levels of several TCA-cycle intermediates (2-oxoglutarate, citrate, cis-aconitate, and fumarate) were evident in the samples from *db/db* mice. At 9 weeks, there were no obvious changes in citrate and cis-aconitate levels between the *wildtype* and *db/db* mice. However, their levels were higher during the middle and late stages of DN (Fig. 4A). 2-oxoglutarate and fumarate levels were high at the early stage of DN and were partly restored to control levels during the late stages. Pyruvate, which is produced by glucose metabolism, was reduced during the progression of DN, with a drastic reduction seen at 17 weeks in *db/db* mice. Additionally, lactate levels were increased in *db/db* animals of 9 weeks (Fig. 4B). These changes indicated altered glucose metabolism during the development and progression of DN. Compared with the *wildtype* mice, creatinine levels, which reflect renal function, were lower in the *db/db* mice throughout the experimental period. Allantoin, 1-methylnicotinamide (MNA), and hippurate were altered in an age-dependent manner. For example, at the age of 13 weeks, hippurate levels were elevated and reached a maximum at 17 weeks in the *db/db* mice, suggesting a strong correlation between hippurate concentration and progression of the disease (Fig. 4C). The lipid byproducts, acetate, 3-hydroxybutyrate (3-HB), and acetoacetate, were reduced in the *db/db* mice throughout the experimental period, suggesting reduced lipid metabolism in DN (Fig. 4D).

### Pattern recognition and quantitative analysis of renal metabolites

**Figure 5:**
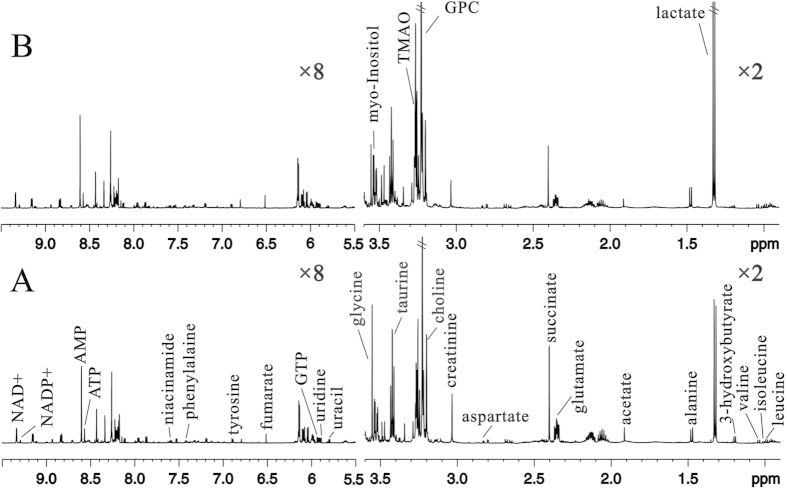
NMR spectra of kidney samples. Representative ^1^H NMR spectra of kidney samples obtained from the *wildtype* mice (**A**) and *db/db* mice (**B**), respectively.

**Figure 6:**
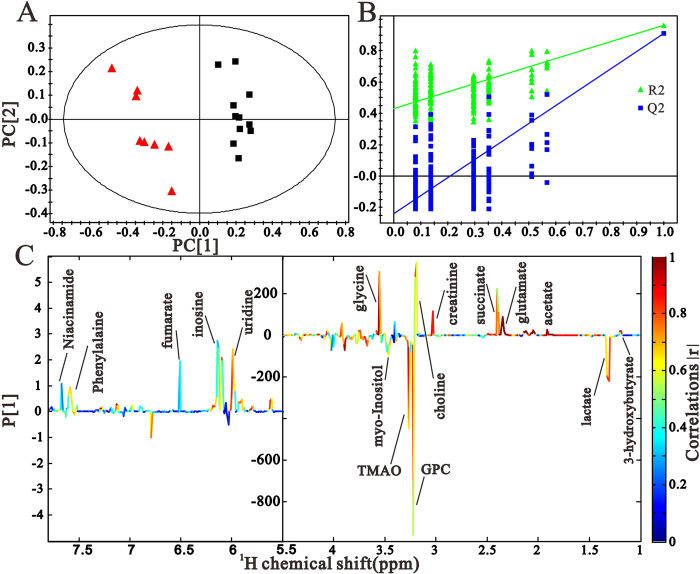
Pattern recognition analysis of kidney samples. The PLS-DA score plot (**A**) and validation plot (**B**) based on the ^1^H NMR spectra of kidney samples obtained from the *wildtype* mice (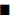) and *db/db* mice (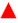). The coefficient-coded loading plot (**C**) corresponding to PLS-DA revealing the metabolites with large intensities responsible for the discrimination of the corresponding score plots.

Representative ^1^H-NMR spectra and peak assignments of the metabolites of the kidney tissue extracts obtained from 17-week-old *db/db* mice are shown in Fig. 5. Similar to that of the urinary metabolites, the PLS-DA score plot of the renal tissue samples (Fig. 6A) from the 17-week-old *db/db* mice and *wildtype* mice showed clear differences along the PC1 direction. The validation plot of permutation tests showed that the PLS-DA model built for *db/db* mice and *wildtype* mice was robust and credible (Fig. 6B). The corresponding loading plot (Fig. 6C) indicated that the variables corresponding to 3-HB, lactate, acetate, glutamate, succinate, creatinine, choline, glycerophosphocholine (GPC), trimetlylamine oxide (TMAO), myo-inositol, glycine, uridine, inosine, fumarate, phenylalaine, and niacinamide were responsible for the separation.

Table 2 shows the relative integrals of metabolites in kidney extracts of the *db/db* and *wildtype* mice. In addition to the urine samples with holistic metabolic characteristics, the metabolic alterations in the kidney tissue shed light on the pathogenic factors involved in DN. Similar to the urinary metabolites, there was higher levels of lactate, and lower levels of acetate, 3-HB, and creatinine in the kidney of *db/db* mice than that of the *wildtype* mice. In addition, reduced levels of glutamate, glycine, tyrosine, uracil, uridine, guanosine triphosphate (GTP), and choline, and significantly increased levels of myo-inositol and TMAO were observed in *db/db* mice.

### Correlation analysis of urinary and renal metabolites

**Figure 7:**
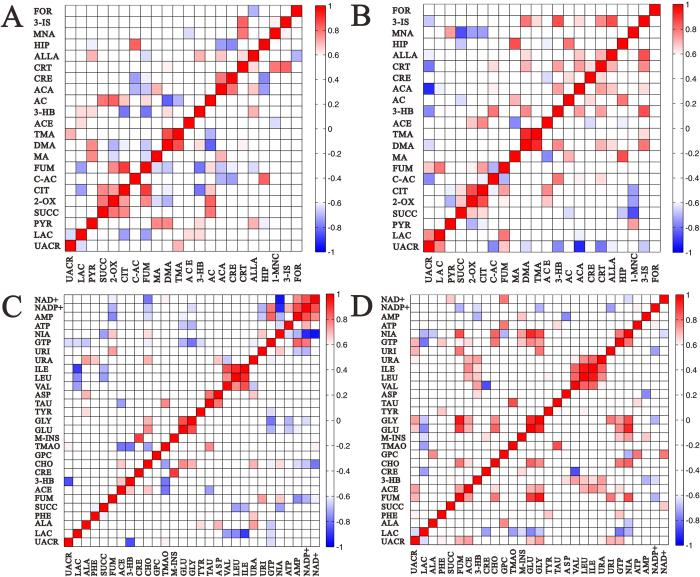
Correlation analysis of urinary and renal metabolites. Pearson’s correlations of UACR and quantities of the metabolites determined from 17-week-old mice urine samples (**A**, *wildtype* mice; **B**, *db/db* mice) and kidney samples (**C**, *wildtype* mice; **D**, *db/db* mice). Red and blue represent positive and negative correlations, respectively, the colour scale represents Pearson’s correlation coefficients. Keys: UACR, urinary albumin to creatinine ratio; LAC, lactate; PYR, pyruvate; SUCC, succinate; 2-OX, 2-oxoglutarate; CIT, citrate; C-AC, cis-aconitate; FUM, fumarate; Ma, methylamine; DMA, dimethylamine; TMA, trimethylamine; ACE, acetate; 3-HB, 3-hydroxybutyrate; AC, acetone; ACA, acetoacetate; CRE, creatine; CRT, creatinine; ALLA, allantion; HIP, hippurate; MNA, 1-methylnicotinamide; 3-IS, 3-indoxylsulfate; FOR, formate; ALA, alanine; PHE, phenylalanine; CHO, choline; M-INS, myo-inositol; GLU, glutamate; GLY, glycine; TYR, tyrosine; TAU, taurine; ASP, aspartate; VAL, valine; LEU, leucine; ILE, isoleucine; URA, uracil; URI, uridine; GTP, guanosine triphosphate; NIA, niacinamide.

To investigate the relationship among the metabolites, their levels in the urine and kidney samples from 17-week-old *wildtype* and *db/db* mice were correlated separately using the Pearson’s correlation (Fig. 7). UACR is a well-known marker of kidney damage and dysfunction. Analysis of the correlation between UACR and identified metabolites can be used to screen for specific biomarkers. Remarkably, the UACR showed a negative correlation with cis-aconitate, dimethylamine (DMA), 3-HB, acetoacetate, creatinine, allantoin, and a positive correlation with fumarate in the urine samples of *db/db* mice. In addition, lactate levels were positively correlated with UACR in the urine samples of *db/db* mice, whereas a negative correlation was observed in the *wildtype* mice (Fig. 7A,B). In the kidney tissue samples of *db/db* mice, UACR was positively correlated with phenylalanine, fumarate, acetate, glutamate, glycine, uridine, and GTP, and negatively correlated with lactate. No such correlation was evident in the *wildtype* mice (Fig. 7C,D). These metabolites, which showed significant correlation with UACR in the *db/db* mice, could serve as potential biomarkers for assessing the progression of DN.

A correlation analysis of the urinary and kidney tissue metabolites from 17-week-old mice was performed to gain insights into the pathogenic characteristics and pathways involved in DN. Positive correlation indicated the relationship of the metabolites with certain pathways, whereas a negative correlation suggested the metabolites may be of different pathways. For example, in the urinary metabolites of *db/db* mice, positive correlations of cis-aconitate with citrate and fumarate, DMA with allantoin and 3-indoxylsulfate, 3-HB and acetoacetate with cis-aconitate, creatinine and 3-indoxylsulface, and hippurate with methylamine and acetone were evident. Further, hippurate from the urine samples of *db/db* mice was negatively correlated with succinate, and acetate (Fig. 7A,B). No such correlations were observed in the urinary metabolites of *wildtype* mice.

Significant correlations were also observed among the metabolites from the kidney tissue samples of *db/db* mice (Fig. 7C,D). Phenylalanine positively correlated with tyrosine. Lactate negatively correlated with creatine, TMAO, myo-inositol, glutamate and glycine. Glutamate and glycine were positively correlated with fumarate, acetate, and GTP, and negatively correlated with lactate. The correlations among the metabolites in *db/db* mice suggested DN-specific alterations in specific metabolic pathways.

### Disturbed metabolic pathways in *db/db* mice

**Figure 8:**
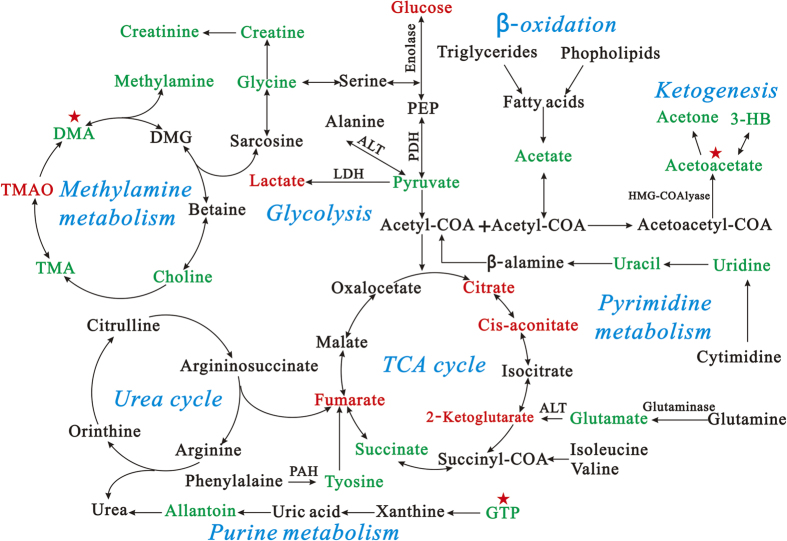
Disturbed metabolic pathways related to pathogenic process of diabetic nephropathy. The metabolite changes detected by ^1^H NMR urine and kidney analysis and the pathway referenced to the KEGG database show the interrelationship of the identified metabolic pathways involved in the *db/db* mice. Metabolites in red and green represent increase and decrease in levels, respectively, compared with *wildtype* mice. Stars represent the potential targets of drugs for diabetic nephropathy.

The pathway analysis, including the analysis of TCA cycle, glycolysis, methylamine metabolism, purine metabolism, pyrimidine metabolism, and fatty acid β-oxidation, was performed using the KEGG database (http://www.genome.jp/kegg/pathway.html). Results showing alterations in metabolic pathways during the progression of DN are summarized in Fig. 8. The reduced concentrations of methylamine, DMA, TMA, and choline in the *db/db* mice suggested the down-regulation of the methylamine pathway. The lower levels of 3-HB, acetone, acetoacetate, and acetate indicated reduced or slow fatty acid β-oxidation in the *db/db* mice. These metabolic changes and the associated pathways provide insights into the mechanisms involved in the development and progression of DN.

## Discussion

DN is a serious complication that develops in a significant proportion of patients with diabetes^11^. Although various factors have been found to influence the onset and progression of DN, the global metabolic alterations associated with renal injury remained poorly characterized. In this study, we used ^1^H-NMR and correlative analysis to identify the characteristic metabolic alterations and pathways associated with the development and progression of DN in the urine and renal tissue samples of *db/db* mice. The key enzymes and proteins involved in the pathways identified as playing a role in the development and progression of the disease could serve as potential drug targets.

### Renal dysfunction and damages in the *db/db* mice

The reduced creatinine levels and elevated UACR values observed in the urine samples of 9- to 13-week-old *db/db* mice confirmed impaired renal function. Creatinine, an indicator of glomerular filtration rate, is formed exclusively from creatine in the body^14^. Creatinine levels were lower in the urine samples collected at five different time-points from the *db/db* mice, indicating renal dysfunction, mild or severe, throughout the experimental period. Moreover, the gradual increase in UACR, a reliable and convenient measure of renal dysfunction^15^, observed in the *db/db* mice indicates the progression of DN with age. These results are consistent with the changes in renal morphology revealed by histological staining with HE and PAS, which showed clear glomerulosclerosis lesions in 17-week-old *db/db* mice, an indication of renal dysfunction and tissue damage seen during the late stages of DN.

### Candidate biomarker screening and identification

Our NMR-based metabonomic analysis revealed several metabolic changes in the urine samples of *db/db* mice. These changes included higher levels of 2-oxoglutarate, citrate, cis-aconitate, fumarate, lactate, hippurate, and MNA, and lower levels of pyruvate, methylamine, creatinine, allantoin, acetate, 3-HB, and acetoacetate. Additionally, analysis of the temporal changes in metabolites revealed that the metabolite levels in urine, especially those of 2-oxoglutarate, citrate, cis-aconitate, fumarate, pyruvate, allantoin, hippurate, and MNA, underwent age-dependent changes during the progression of DN. These characteristic changes could serve as potential biomarkers for the diagnosis of DN. Finally, correlation analysis of urinary metabolites and UACR value of 17-week-old *db/db* mice further identified cis-aconitate, and allantoin as strong candidate biomarkers for the diagnosis of DN.

Hippurate is generally detected in urine and its concentration reflects the microbial activity and composition of the gut^6^. Increased levels of hippurate found in the urine of *db/db* mice from 13-weeks to 17-weeks of age suggest that there are alterations in the gut microbiota. Urinary hippurate has been reported to be an early biomarker of nephrotoxicity resulting from nephrotoxin administration in rats^16^. High urinary excretion of hippurate has also been found to correlate with reduced glomerular filtration rate and tubular reabsorption^17^. Therefore, elevated hippurate levels in *db/db* mice also reflect altered glomerular filtration. Further the age-dependent changes in hippurate levels suggest that hippurate may be a potential indicator of the progression of DN. However, we did not observe a correlation between urinary hippurate and UACR in *db/db* mice. Therefore, hippurate might not be a reliable biomarker of DN.

Because allantoin is not reabsorbed in the proximal tubule, urinary allantoin concentration is thought to accurately reflect glomerular filtration rate^18^. Therefore, the decreased level of allantoin in urine samples at 17 weeks is an indicator of late stage of DN in the *db/db* mice at this time point. The negative correlation between allantioin and UACR, and the age-dependent alterations suggest that allantoin could be used as a biomarker for the diagnosis of DN. Additional pharmacological or biochemical studies are needed to test the specificity and sensitivity of these metabolites as biomarkers of DN.

### Glucose metabolism

Correlations among the different metabolites provide valuable information regarding their source metabolic pathways, whereas correlations between metabolite levels and UACR provide information regarding the relationship of the metabolites to the pathogenesis of DN. Together, these analyses could provide clues to the nature of enzymes and other proteins, potential drug targets, involved in the development and progression of DN.

Results of our analysis showed that there were increased concentrations of the TCA cycle intermediates such as citrate, 2-oxoglutarate, cis-aconitate, and fumarate in the majority of the urine samples of *db/db* mice. This might reflect either systemic stress caused by hyperglycemia or local effects on kidney tubular transport and impairment of mitochondrial function^19^. Compared with 9-week-old mice, significantly higher levels of citrate and cis-aconitate were present in the *db/db* mice at later stages. This result suggests that TCA cycle is accelerated during the middle and late stages of DN. Notably, among the TCA cycle intermediates, only succinate level showed a decline. Therefore, it appears that pathways other than TCA cycle, such as specific succinate transporters, are responsible for the changes in succinate levels and may play a role in determining the disease status^20^.

The elevated level of lactate found in the urine and renal tissue samples of *db/db* mice is indicative of increased glycolysis. These metabolic features of TCA cycle and increased glycolysis seen in *db/db* mice indicate that glucose metabolism is involved in the development of DN. Additionally, the significant correlations of cis-aconitate and fumarate with UACR, which were absent in the corresponding control mice, indicate that alterations in glucose metabolism contribute to the development and progression of DN.

### Methylamine metabolism

Some methylamine metabolites are important non-perturbing renal osmolytes produced through the degradation of dietary choline to TMA and its di- and monoamine metabolites by the action of gut microflora^21^. The decreased urinary concentrations of methylamine and DMA, and the renal concentration of choline in the *db/db* mice suggest the downregulation of methylamine pathway in DN. Their decline could be seen as due to osmotic compensation for elevated blood glucose concentrations or might indicate renal papillary dysfunction in DN^22^. However, the renal concentration of TMAO showed a tendency different from that of other metabolites of the methylamine metabolism pathway. It is likely that other pathways contributed to the contradictions and further studies are needed to understand the precise nature of these contributing factors.

We found that DMA negatively correlated with UACR in the urine of *db/db* mice, which suggests that DMA plays a key role in the progression of DN. DMA circulates in the body and is excreted through urine. Asymmetric dimethylarginine (ADMA), an endogenous inhibitor of nitric oxide (NO) synthase, is a major precursor of DMA^23^. ADMA is hydrolyzed to DMA and L-citrulline by dimethylarginine dimethylaminohydrolase (DDAH). Therefore, pharmacological modulation of DDAH activity has been proposed as a method for manipulating endogenous DMA concentrations and regulating the production of nitric oxide (NO) in conditions where they have been shown to contribute to illnesses^24^. It has been reported that DDAH plays an important role in childhood hypercholesterolemia^25^ and idiopathic pulmonary fibrosis^26^. The anti-diabetic drug metformin has been shown to effectively modulate the metabolism of asymmetric dimethylarginine in experimental liver injury^27^. The therapeutic action of relaxin during hypertension is also dependent on the modulation of endogenous DMA levels and DDAH activity^28^. Thus, DDAH plays a key role in the maintenance of vascular homeostasis. Together with these earlier findings, our results suggest that inhibition of DDAH might be a promising therapeutic strategy for the treatment of patients suffering from DN.

### Fatty acid metabolism

3-HB and acetoacetate are products of ketone bodies and fatty acid metabolism in liver mitochondria^29^. Our NMR analysis of the urine samples revealed decreased levels of 3-HB and acetoacetate in *db/db* mice throughout the course of the experiment, suggesting a reduced activity of ketogenesis pathway or the presence of mitochondrial dysfunction in DN. Additionally, we found that 3-HB positively correlated with acetoacetate in the urine of *db/db* mice, whereas such a correlation was absent in the control mice. These changes suggest significantly increased correlation between ketone bodies metabolism and the pathological state of DN. We also found that 3-HB and acetoacetate displayed significant negative correlations with UACR, indicating that these metabolites are associated with the pathogenesis of DN. In hepatic cells, 3-hydroxy-3-methylglutaryl-CoA lyase (HMG-CoA lyase) functions as a rate-limiting enzyme that catalyzes the cleavage of HMG-CoA to acetoacetate, which is partly reduced to form 3-HB^30^. Inhibition of HMG-CoA lyase activity by dietary polyphenols has been proposed as a therapeutic strategy for preventing ketoacidosis^31^. Therefore, ketogenesis or HMG-CoA lyase or both may be potential targets for drugs in the treatment of DN.

Acetate is another important product of fatty acid β-oxidation and its decline in urine and kidney of *db/db* mice suggests significantly reduced fatty acid β-oxidation in DN. Together, these results are indicative of impaired fatty acid metabolism in *db/db* mice.

### Glycogenic amino acid metabolism

Compared with that in the control mice, concentrations of glutamate, tyrosine, and glycine were lower in the kidney of *db/db* mice, suggesting that protein and amino acid metabolisms were impaired in diabetic mice. Both glycine and glutamate levels showed significant negative correlations with lactate in DN mice. Glycine and glutamate are important glycogenic amino acids. Thus, these negative correlations indicate enhancement of gluconeogenesis pathway during the pathogenesis of DN. Our analysis showed that glycine and glutamate positively correlated with UACR in the *db/db* mice, whereas such correlations were absent in the control mice. These results confirm that gluconeogenesis is closely related to the pathogenesis of DN. Ameliorating glucose metabolism by regulating gluconeogenesis is one of the important strategies against diabetic complications.

### Nucleotide metabolism

1-methylnicotinamide (MNA) is involved in the tryptophan-NAD pathway, which supplies pyridine nucleotides to the liver^32^. MNA has been suggested as urinary and plasma biomarkers of peroxisome proliferation in rats^33^. Because it affects oxidative and glycooxidative processes, MNA has been considered for potential applications in the prevention and treatment of various complications accompanying diabetes mellitus^34^. We found increased concentration of MNA in the urine of *db/db* mice, indicating the possible protective effects of the metabolite in DN. However, our results showed that there was no correlation between MNA and other important metabolites in the *db/db* mice. Therefore, MNA itself might not be regarded as a suitable therapeutic drug for DN.

The reduced levels of uracil, uridine, and GTP in the kidney of *db/db* mice suggest a disturbance in the nucleotide metabolism during the development of DN. The highly positive correlations of uridine and GTP levels with UACR indicate that nucleotide metabolism is closely related to the pathogenesis of DN. As a fundamental unit of all forms of life, GTP serves as a key donor substrate for protein and lipid phosphorylation^35^. Different pathological states are accompanied by changes in erythrocyte GTP concentrations. GTP cyclohydrolase I (GTPCH I) is the rate-limiting enzyme in the biosynthesis of BH4 from GTP, an essential cofactor for nitric oxide synthase and aromatic amino acid hydroxylase^36^. Mutation of GTPCH I has been identified as showing some therapeutic promises against cardiovascular or neurological diseases^37^. Therefore, pharmacological modulation of GTPCH I activity or GTP levels might be a promising new strategy for the treatment of DN.

### Changes in other metabolites

Myo-inositol is a component of cell membranes and plays an important role in cell morphogenesis and cytogenesis, lipid synthesis, structure of cell membranes and cell growth^38^. The observed increase in the myo-inositol concentration in the kidney extracts of *db/db* mice indicates tubular dysfunction and renal cell stress under hyperglycemia, likely due to the downregulated expression of the myo-inositol oxygenase in *db/db* mice^39^. Myo-inositol has been suggested to play an important role in the etiology of diabetes mellitus, particularly with respect to the progression of DN, and its modulation has been shown to have a beneficial effect in restoring the impaired motor nerve conduction velocity and disruption of nerve structural elements^40^. However, our analysis showed no correlation of the metabolite with UACR. Further studies are needed to examine the possible role of myo-inositol in the development and progression of DN.

## Conclusions

In summary, we integrated information obtained from the ^1^H NMR-based metabonomic analysis of urine and kidney samples of *db/db* and *wildtype* mice to characterize the systemic metabolic changes during the development of DN. Combined metabolic alterations and correlation analysis identified cis-aconitate and allantoin as potential biomarkers of DN. Also the results revealed that several enzymes, including DDAH, HMG-CoA lyase, and GTPCH I could be targeted for intervention. Together, the present results pave a way for elucidating the mechanisms underlying the development and progression of DN, provide new avenues for the diagnosis of DN and present potential targets for drug discovery.

## Methods

### Animals

BKS.Cg-m^+/+^ Leprdb/J *db/db* (n = 10, male) mice that are deficient for leptin receptor (Lepr^−^/Lepr^−^) were used as DN models, while genetically normal C57BLKS/J *wildtype* mice (n = 15, male) were used as control^41^. The animals were obtained from Model Animal Research Center of Nanjing University, and kept in a SPF colony of the Laboratory Animal Center of Wenzhou Medical University with regulated temperature and humidity and a 12/12 h light–dark cycle for 10 weeks. During the entire experiment, mice were fed with standard chow and tap water. All animals received care in accordance to the ‘Guide for the Care and Use of Laboratory Animals’. Procedures using mice were approved by the Institutional Animal Care and Use Committee of Wenzhou Medical College (document number: wydw2012-0083).

### Urine and kidney sample collection

Urine samples were collected for 12 h after fasting at 9-, 11-, 13-, 15- and 17-weeks of age in metabolic cages individual. The urine samples were collected over ice into 0.1 ml of 1% sodium azide solution for avoiding bacterial contamination and then centrifuged for 10 min at 4 °C. The supernatant was stored at −80 °C until measurement. The body weight of the mice was measured weekly and the fasting blood glucose concentration was measured using a tail nick and glucometer (B/BRAUN omnitest plus). Animals were killed by cervical dislocation at the age of 17-weeks, and their renal tissues were dissected, immediately snap-frozen in liquid nitrogen, and stored at −80 °C freezer until NMR experiments.

### Clinical biochemistry and histological analysis

Clinical chemistry analysis of the urine was carried out on an automatic biochemistry analyzer (Mindray BS-300) using appropriate kits with the following parameters: CRE, UACR. The renal tissues were rapidly dissected, fixed overnight in 10% buffered formalin, and then embedded in paraffin, made into 4 mm sections and further stained with HE and PAS.

### Preparation of samples and acquisition of ^1^H-NMR spectra

Before NMR analysis, urine samples were thawed, and 500 μL aliquots were mixed with 50 μL D_2_O containing TSP (0.36 mg/mL) and 300 μL of phosphate buffer (0.2 M Na_2_HPO_4_/NaH_2_PO_4_, pH 7.4) to minimize variations in pH^42^. D_2_O provided a field-frequency lock, and TSP was used as the chemical shift reference. The mixtures were centrifuged to remove the precipitates, and then 500 μL of the supernatant was transferred to 5-mm NMR tubes. All NMR spectra were recorded at 25 °C on a Bruker AVANCE III 600 MHz NMR spectrometer equipped with a triple resonance probe and a z-axis pulsed field gradient. ^1^H NMR spectra were acquired using a one-dimensional NOESY pulse sequence with water suppression during the relaxation delay of 4 s and a mixing time of 150 ms. 128 free induction decays were collected into 32 K data points with a spectral width of 12 000 Hz, an acquisition time of 2.66 s. FID was zero-filled to 64 K prior to Fourier transformation.

The frozen renal tissue was weighed and put into a centrifuge tube. Ice-cold methanol (4 mL/g) and distilled water (0.85 mL/g) was added into the tube, homogenized at 4 °C after thawing and mixed by vortex. Chloroform (2 mL/g) and distilled water (2 mL/g) was added into the tube and mixed again. After the sample tubes were kept on ice for 15 min, the homogenate was centrifuged at 1 000 g for 15 min at 4 °C. The supernatant was extracted and lyophilized for about 24 h. Before recording of the NMR spectra, the tissue extracts were resuspended in 500 μL D_2_O containing TSP (0.25 mM), and then after centrifugation the supernatant was transferred to 5 mm NMR tubes. ^1^H-NMR spectra were acquired at 25 °C on a Bruker AVANCE III 600 MHz NMR spectrometer equipped with a triple resonance probe and a z-axis pulsed field gradient. A one-dimensional ZGPR pulse sequence was used to achieve satisfactory water suppression in aqueous extracts. For each sample, the relaxation delay was 6.0 s and the acquisition time was 2.66 s per scan. 128 transients were collected into 64 K data points with a spectral width of 12 000 Hz.

### Data reduction and multivariate pattern recognition analysis

All NMR spectra were phased and baseline corrected, and then data-reduced to 1100 integrated regions of 0.01 ppm width corresponding to the region of δ 10 to −1 using the Topspin 2.1 software package for multivariate pattern recognition analysis. And another data-reduced to 7334 integrated regions of 0.0015 ppm width corresponding to the region of δ 10 to −1 for quantitative analysis. For NMR spectra recorded in kidney extracts, the region of about δ 4.69–5.04 was removed to eliminate artifacts related to the residual water resonance. The region of the urine spectra associated with residual water and urea (4.71–5.06 and 5.72–5.94 ppm) were removed. And due to the presence of conspicuous glucose metabolite, the resonances region of glucose of all spectra were also excluded^19^,^43^. The remaining spectral segments were normalized to the total sum of the spectral intensity to compensate for variations in total sample volume. The normalized integral values were then subjected to multivariate pattern recognition analysis using the SIMCA-P + V12.0 software package (Umetrics, Umea, Sweden).

PLS-DA was carried out for class discrimination and biomarker identification. Data were visualized by plotting the scores of the first two principal components (PC1 and PC2) to provide the most efficient 2D representation of the information, where the position of each point along a given axis in the scores plot was influenced by variables in the same axis in the loading plot^44^. PLS-DA revealed differences in the urine and kidney composition of different groups, which were necessary to eliminate outliers and enhance the quality of the PCA model. Leave one-out cross validation and permutation tests (200 cycles) were conducted to measure the robustness of the model obtained because of the small number of samples^45^. The scores and loading plots complemented each other. PLS-DA loading plots were used to identify the metabolites responsible for the separation of groups^46^. Significant differences among metabolites in PLS-DA were assessed by the absolute value of the correlation coefficient, |r|, which was calculated in a Java environment.

### Statistical analysis

A system statistical metabolic correlation analysis was further applied to display the relationships between certain metabolite integrals, as described previously^43^. Metabolite intensities relative to the sum of the total spectral integral were used as variables and Pearson’s correlation coefficient was calculated among those variables in the above-mentioned Java environment. An absolute value of correlation coefficient |r| > 0.6 (*P* < 0.05) indicates a statistically significant relationship. Positive values masked in the pixel map are shown with red colors and negative values are indicated with blue colors.

The normalized integral values were then input into the software of SPSS for Windows (Version 13.0, SPSS Inc., USA) for the statistical analysis. A two-tailed unpaired t-test was applied in order to detect significant differences in selected signals between two groups. A calculated *P*-value of less than 0.05 was considered statistically significant. Data were presented as mean ± SE.

## Additional Information

**How to cite this article**: Wei, T. *et al.* Metabonomic analysis of potential biomarkers and drug targets involved in diabetic nephropathy mice. *Sci. Rep.* **5**, 11998; doi: 10.1038/srep11998 (2015).

## Figures and Tables

**Table 1:** Morphometric and metabolic parameters.

	9-wk	11-wk	13-wk	15-wk	17-wk
Body weight, g					
* wildtype* mice	22.59 ± 0.35	19.91 ± 0.28	20.35 ± 0.31	20.88 ± 0.32	22.21 ± 0.34
* db/db* mice	41.14 ± 0.96^**^	40.34 ± 1.09^**^	42.35 ± 1.14^**^	45.36 ± 1.24^**^	50.85 ± 0.96^**^
Serum glucose, mmol/L					
* wildtype* mice	6.31 ± 0.35	4.92 ± 0.33	5.20 ± 0.34	6.56 ± 0.89	4.93 ± 0.11
* db/db* mice	17.08 ± 0.88^**^	20.72 ± 2.15^**^	17.33 ± 1.00^**^	12.94 ± 0.83^**^	10.49 ± 0.35^**^
CRE, mg/dL					
* wildtype* mice	10.52 ± 0.48	14.51 ± 1.57	14.35 ± 1.81	16.95 ± 1.89	19.14 ± 2.33
* db/db* mice	6.02 ± 0.81^**^	4.68 ± 0.55^**^	4.50 ± 0.53^**^	3.80 ± 0.64^**^	3.48 ± 0.71^**^
UACR, mg/mmol					
* wildtype* mice	56.57 ± 5.92	83.77 ± 12.15	77.82 ± 10.86	65.76 ± 5.93	65.13 ± 8.95
* db/db* mice	95.47 ± 6.50^**^	135.00 ± 18.57^*^	131.37 ± 18.57^*^	138.28 ± 19.68^**^	153.62 ± 27.06^*^

Values are expressed as mean ± SE; Keys: CRE, urine creatinine; UACR, urinary albumin to creatinine ratio; ^*^P < 0.05, ^**^P < 0.01 compared with age-matched *wildtype* mice.

**Table 2:** Summary of the potential biomarkers in kidney extracts from *db/db* mice and *wildtype* mice.

δ^1^H(ppm)	metabolites	metabolism pathway	*wildtype* mice	*db/db* mice
1.342	Lactate	Glycolysis	38.78 ± 0.76	53.27 ± 2.42^**^
1.919	Acetate	Fatty acids β-oxidation	3.31 ± 0.09	1.93 ± 0.09^**^
1.206	3-hydroxybutyrate	Ketogenesis	3.39 ± 0.20	1.52 ± 0.05^**^
3.037	Creatinine	Serinolysis	7.59 ± 0.22	4.50 ± 0.28^**^
3.204	Choline	Methylamine metabolism	21.12 ± 1.02	14.12 ± 0.87^**^
3.275	TMAO	Methylamine metabolism	7.52 ± 0.13	9.41 ± 0.28^**^
3.545	myo-Inositol	Lipid metabolism	27.45 ± 0.26	28.47 ± 0.34^*^
2.372	Glutamate	Amino acid metabolism	22.23 ± 0.42	12.68 ± 0.64^**^
3.557	Glycine	Amino acid metabolism	16.05 ± 0.37	8.10 ± 0.52^**^
6.909	Tyrosine	Amino acid metabolism	0.44 ± 0.01	0.39 ± 0.01^*^
5.809	Uracil	Pyrimidine metabolism	0.22 ± 0.01	0.16 ± 0.01^**^
5.92	Uridine	Pyrimidine metabolism	0.88 ± 0.02	0.73 ± 0.04^**^
5.94	GTP	Purine metabolism	0.55 ± 0.01	0.47 ± 0.01^**^

Values are expressed as mean ± SE; Keys: TMAO, trimetlylamine oxide; GTP, guanosine triphosphate; ^*^P < 0.05, ^**^P < 0.01 compared with age-matched *wildtype* mice.

## References

[b1] Shao N. *et al.* Relationship between Oxidant/Antioxidant Markers and Severity of Microalbuminuria in the Early Stage of Nephropathy in Type 2 Diabetic Patients. J. Diabetes Res. 2013, 232404 (2013). 23671859 10.1155/2013/232404 PMC3647557

[b2] Kanwar Y. S., Sun L., Xie P., Liu F. Y. & Chen S. A glimpse of various pathogenetic mechanisms of diabetic nephropathy. Annu. Rev. Pathol. 6, 395–423 (2011). 21261520 10.1146/annurev.pathol.4.110807.092150 PMC3700379

[b3] Shockcor J. P. & Holmes E. Metabonomic applications in toxicity screening and disease diagnosis. Curr. Top. Med. Chem. 2, 35–51 (2002). 11899064 10.2174/1568026023394498

[b4] Nicholson J. K. Global systems biology, personalized medicine and molecular epidemiology. Mol. Syst. Biol. 2, 52 (2006). 17016518 10.1038/msb4100095 PMC1682018

[b5] Nicholson J. K., Lindon J. C. & Holmes E. ‘Metabonomics’: understanding the metabolic responses of living systems to pathophysiological stimuli via multivariate statistical analysis of biological NMR spectroscopic data. Xenobiotica 29, 1181–1189 (1999). 10598751 10.1080/004982599238047

[b6] Psihogios N. G., Gazi I. F., Elisaf M. S., Seferiadis K. I. & Bairaktari E. T. Gender-related and age-related urinalysis of healthy subjects by NMR-based metabonomics. NMR Biomed. 21, 195–207 (2008). 17474139 10.1002/nbm.1176

[b7] Zhao L. *et al.* ^1^H-NMR-based metabonomic analysis of metabolic profiling in diabetic nephropathy rats induced by streptozotocin. Am. J. Physiol. Renal Physiol. 300, F947–956 (2011). 21228107 10.1152/ajprenal.00551.2010

[b8] Diao C. *et al.* Systemic and characteristic metabolites in the serum of streptozotocin-induced diabetic rats at different stages as revealed by a ^1^H-NMR based metabonomic approach. Mol. Biosyst. 10, 686–693 (2014). 24448714 10.1039/c3mb70609e

[b9] Guan M. *et al.* Systemic perturbations of key metabolites in diabetic rats during the evolution of diabetes studied by urine metabonomics. PLoS One 8, e60409 (2013). 23573250 10.1371/journal.pone.0060409 PMC3616076

[b10] Li M. *et al.* GC/TOFMS analysis of metabolites in serum and urine reveals metabolic perturbation of TCA cycle in db/db mice involved in diabetic nephropathy. Am. J. Physiol. Renal Physiol. 304, F1317–1324 (2013). 23467425 10.1152/ajprenal.00536.2012

[b11] Liu J. *et al.* ^1^H NMR-based metabonomic analysis of serum and urine in a nonhuman primate model of diabetic nephropathy. Mol. Biosyst. 9, 2645–2652 (2013). 24228270 10.1039/c3mb70212j

[b12] Sharma K., McCue P. & Dunn S. R. Diabetic kidney disease in the db/db mouse. Am. J. Physiol. Renal Physiol. 284, F1138–1144 (2003). 12736165 10.1152/ajprenal.00315.2002

[b13] Hu Y. *et al.* Functional annotations of diabetes nephropathy susceptibility loci through analysis of genome-wide renal gene expression in rat models of diabetes mellitus. BMC Med. Genomics 2, 41 (2009). 19586551 10.1186/1755-8794-2-41 PMC2717999

[b14] Akira K., Masu S., Imachi M., Mitome H. & Hashimoto T. A metabonomic study of biochemical changes characteristic of genetically hypertensive rats based on ^1^H NMR spectroscopic urinalysis. Hypertens Res. 35, 404–412 (2012). 22089538 10.1038/hr.2011.182

[b15] Teimoury A., Iraj B., Heidari-Beni M., Amini M. & Hosseiny S. M. Why 24-h Urine Albumin Excretion Rate Method Still is Used for Screening of Diabetic Nephropathy in Isfahan Laboratories? Int. J. Prev. Med. 5, 341–347 (2014). 24829719 PMC4018644

[b16] Boudonck K. J. *et al.* Discovery of metabolomics biomarkers for early detection of nephrotoxicity. Toxicol. Pathol. 37, 280–292 (2009). 19380839 10.1177/0192623309332992

[b17] Zuppi C. *et al.* Proton nuclear magnetic resonance spectral profiles of urine from children and adolescents with type 1 diabetes. Clin. Chem. 48, 660–662 (2002). 11901070

[b18] Briggs J. P., Levitt M. F. & Abramson R. G. Renal excretion of allantoin in rats: a micropuncture and clearance study. Am. J. Physiol. 233, F373–381 (1977). 920807 10.1152/ajprenal.1977.233.5.F373

[b19] Salek R. M. *et al.* A metabolomic comparison of urinary changes in type 2 diabetes in mouse, rat, and human. Physiol. Genomics 29, 99–108 (2007). 17190852 10.1152/physiolgenomics.00194.2006

[b20] Peti-Peterdi J. High glucose and renin release: the role of succinate and GPR91. Kidney Int. 78, 1214–1217 (2010). 20861827 10.1038/ki.2010.333

[b21] Messenger J., Clark S., Massick S. & Bechtel M. A review of trimethylaminuria: (fish odor syndrome). J. Clin. Aesthet. Dermatol. 6, 45–48 (2013). 24307925 PMC3848652

[b22] Zhao L. C. *et al.* A metabonomic comparison of urinary changes in Zucker and GK rats. J. Biomed. Biotechnol. 2010, 431894 (2010). 20981252 10.1155/2010/431894 PMC2963802

[b23] Chobanyan K., Mitschke A., Gutzki F. M., Stichtenoth D. O. & Tsikas D. Accurate quantification of dimethylamine (DMA) in human plasma and serum by GC-MS and GC-tandem MS as pentafluorobenzamide derivative in the positive-ion chemical ionization mode. J. Chromatogr. B. Analyt. Technol. Biomed. Life Sci. 851, 240–249 (2007). 10.1016/j.jchromb.2007.03.006 17400039

[b24] Brinkmann S. J., de Boer M. C., Buijs N. & van Leeuwen P. A. Asymmetric dimethylarginine and critical illness. Curr. Opin. Clin. Nutr. Metab. Care 17, 90–97 (2014). 24281375 10.1097/MCO.0000000000000020

[b25] Chobanyan-Jurgens K. *et al.* Increased asymmetric dimethylarginine (ADMA) dimethylaminohydrolase (DDAH) activity in childhood hypercholesterolemia type II. Amino Acids 43, 805–811 (2012). 22075968 10.1007/s00726-011-1136-3

[b26] Pullamsetti S. S. *et al.* The role of dimethylarginine dimethylaminohydrolase in idiopathic pulmonary fibrosis. Sci. Transl. Med. 3, 87ra53 (2011). 10.1126/scitranslmed.3001725 21677199

[b27] Bal F. *et al.* Antidiabetic drug metformin is effective on the metabolism of asymmetric dimethylarginine in experimental liver injury. Diabetes Res. Clin. Pract. 106, 295–302 (2014). 25263501 10.1016/j.diabres.2014.08.028

[b28] Sasser J. M., Cunningham M. W. Jr. & Baylis C. Serelaxin reduces Oxidative Stress and Asymmetric Dimethylarginine in Angiotensin II Induced Hypertension. Am. J. Physiol. Renal Physiol. 307, F1355–1362 (2014). 25298524 10.1152/ajprenal.00407.2014 PMC4269693

[b29] Wu H. *et al.* NMR spectroscopic-based metabonomic investigation on the acute biochemical effects induced by Ce(NO_3_)_3_ in rats. J. Inorg. Biochem. 99, 2151–2160 (2005). 16144712 10.1016/j.jinorgbio.2005.07.014

[b30] Fu Z., Runquist J. A., Montgomery C., Miziorko H. M. & Kim J. J. Functional insights into human HMG-CoA lyase from structures of Acyl-CoA-containing ternary complexes. J. Biol. Chem. 285, 26341–26349 (2010). 20558737 10.1074/jbc.M110.139931 PMC2924059

[b31] Nakagawa S., Kojima Y., Sekino K. & Yamato S. Effect of polyphenols on 3-hydroxy-3-methylglutaryl-coenzyme A lyase activity in human hepatoma HepG2 cell extracts. Biol. Pharm. Bull. 36, 1902–1906 (2013). 24292050 10.1248/bpb.b13-00334

[b32] Wolf H. The effect of hormones and vitamin B6 on urinary excretion of metabolites of the kynurenine pathway. Scand. J. Clin. Lab. Invest. Suppl. 136, 1–186 (1974). 4275489

[b33] Connor S. C. *et al.* Development of a multivariate statistical model to predict peroxisome proliferation in the rat, based on urinary ^1^H-NMR spectral patterns. Biomarkers 9, 364–385 (2004). 15764299 10.1080/13547500400006005

[b34] Orszaghova Z. *et al.* Effects of N1-methylnicotinamide on oxidative and glycooxidative stress markers in rats with streptozotocin-induced diabetes mellitus. Redox Rep. 17, 1–7 (2012). 22340509 10.1179/1351000211Y.0000000016 PMC6837363

[b35] Dagher P. C. Apoptosis in ischemic renal injury: roles of GTP depletion and p53. Kidney Int. 66, 506–509 (2004). 15253698 10.1111/j.1523-1755.2004.761_7.x

[b36] Ren J. Hope or hype: The obsession for tetrahydrobiopterin and GTP cyclohydrolase I (GTPCH I) in cardiovascular medicine. J. Cardiothorac. Renal Res. 1, 15–21 (2006).

[b37] Du J. *et al.* Identification of proteins interacting with GTP cyclohydrolase I. Biochem. Biophys. Res. Commun. 385, 143–147 (2009). 19442649 10.1016/j.bbrc.2009.05.026 PMC2737525

[b38] Carlomagno G., De Grazia S., Unfer V. & Manna F. Myo-inositol in a new pharmaceutical form: a step forward to a broader clinical use. Expert Opin. Drug Deliv. 9, 267–271 (2012). 22339497 10.1517/17425247.2012.662953

[b39] Kanwar Y. S. *et al.* Diabetic nephropathy: mechanisms of renal disease progression. Exp. Biol. Med. (Maywood) 233, 4–11 (2008). 18156300 10.3181/0705-MR-134

[b40] Prabhu K. S., Arner R. J., Vunta H. & Reddy C. C. Up-regulation of human myo-inositol oxygenase by hyperosmotic stress in renal proximal tubular epithelial cells. J. Biol. Chem. 280, 19895–19901 (2005). 15778219 10.1074/jbc.M502621200

[b41] Ghosh S. *et al.* Moderate exercise attenuates caspase-3 activity, oxidative stress, and inhibits progression of diabetic renal disease in db/db mice. Am. J. Physiol. Renal Physiol. 296, F700–708 (2009). 19144689 10.1152/ajprenal.90548.2008 PMC2670639

[b42] Xiao C., Hao F., Qin X., Wang Y. & Tang H. An optimized buffer system for NMR-based urinary metabonomics with effective pH control, chemical shift consistency and dilution minimization. Analyst 134, 916–925 (2009). 19381385 10.1039/b818802e

[b43] Zhang S. *et al.* Correlative and quantitative ^1^H NMR-based metabolomics reveals specific metabolic pathway disturbances in diabetic rats. Anal. Biochem. 383, 76–84 (2008). 18775407 10.1016/j.ab.2008.07.041 PMC3867451

[b44] Westerhuis J. A., van Velzen E. J., Hoefsloot H. C. & Smilde A. K. Multivariate paired data analysis: multilevel PLSDA versus OPLSDA. Metabolomics 6, 119–128 (2010). 20339442 10.1007/s11306-009-0185-z PMC2834771

[b45] Weljie A. M., Dowlatabadi R., Miller B. J., Vogel H. J. & Jirik F. R. An inflammatory arthritis-associated metabolite biomarker pattern revealed by ^1^H NMR spectroscopy. J. Proteome Res. 6, 3456–3464 (2007). 17696462 10.1021/pr070123j

[b46] Cloarec O. *et al.* Evaluation of the orthogonal projection on latent structure model limitations caused by chemical shift variability and improved visualization of biomarker changes in ^1^H NMR spectroscopic metabonomic studies. Anal. Chem. 77, 517–526 (2005). 15649048 10.1021/ac048803i

